# Knockdown of serine/threonine-protein kinase 24 promotes tumorigenesis and myeloid-derived suppressor cell expansion in an orthotopic immunocompetent gastric cancer animal model

**DOI:** 10.7150/jca.35821

**Published:** 2020-01-01

**Authors:** Hui-Ping Hsu, Chih-Yang Wang, Pei-Yin Hsieh, Jung-Hua Fang, Yi-Ling Chen

**Affiliations:** 1 Department of Surgery, National Cheng Kung University Hospital, College of Medicine, National Cheng Kung University, Tainan, Taiwan; 2 Department of Biochemistry and Molecular Biology, College of Medicine, National Cheng Kung University, Tainan, Taiwan; 3 Institute of Basic Medical Sciences, College of Medicine, National Cheng Kung University, Tainan, Taiwan; 4 Laboratory Animal Center, College of Medicine, National Cheng Kung University, Tainan, Taiwan; 5 Department of Senior Citizen Service Management, Chia Nan University of Pharmacy and Science, Tainan, Taiwan; 6 Senior Citizen Development Center, Chia Nan University of Pharmacy and Science, Tainan, Taiwan

**Keywords:** gastric cancer, serine/threonine-protein kinase 24, myeloid-derived suppressor cells, germinal center kinase, tumorigenesis, orthotopic model of gastric cancer

## Abstract

A higher incidence of gastric cancer has been found in East Asia compared to the incidence in other regions. Gastric cancer patients have a poor prognosis due to distant metastasis and advanced cancer stages. Tumor escape pathways include the expansion of the immunosuppressive myeloid-derived suppressor cells (MDSCs) in the tumor microenvironment. We have successfully established an orthotopic immunocompetent gastric cancer model in C57BL/6 mice. The cell line is named M12 and was deposited at the Bioresource Collection and Research Center of Taiwan on Sep. 13, 2016 (Patent No. I604054). The orthotopic animal model of gastric cancer has similar biological characteristics as human gastric cancer. Serine/threonine-protein kinase 24** (**STK24) is a member of the germinal center kinase (GCK)-III family. GCKs participate in cancer and immunological disorders. The effects of STK24 in gastric cancer are less well understood. CRISPR (clustered regularly interspaced short palindromic repeats)/Cas9 technology was used to induce a *STK24* genetic knockout at the genomic DNA level in tumor cells. The knockdown of the *STK24* gene increased the tumor growth in an orthotopic model of gastric cancer. The *STK24* gene silencing in tumors induced the expansion of CD11b^+^Ly6C^+^ cells and F4/80^+^ macrophages *in vivo*. To our knowledge, we have developed the first orthotopic transplantable model of gastric cancer in syngeneic inbred mice. Our results further indicate that STK24 is important for immune regulation during the tumorigenesis of gastric cancer.

## Introduction

Animal models are essential research tools for the study of tumor immunity and the development of immunotherapy. Previous experiments with immunocompromised mice for the *in vivo* transplantation of gastric cancer and targeted therapies through immune modification cannot be evaluated. Recently, an orthotopic transplantable model of syngeneic gastric cancer has been developed by our team in immunocompetent inbred mice. Therefore, we utilized these immunocompetent C57BL/6 mice to fully study the cancer immunotherapy of gastric cancer.

Gastric cancer is a common cancer in men and in older adults. The incidence and mortality of gastric cancer is the highest in East Asia 1. Gastric cancer often causes nonspecific symptoms in the early stages. The majority of patients have a poor prognosis due to an advanced cancer stage and the metastatic spread of gastric cancer. The mechanisms of tumor escape include the loss of antigenicity, the loss of immunogenicity and an immunosuppressive microenvironment 2. The interaction of the host immune system and tumor cells creates a tumor microenvironment. Recently, the tumor microenvironment is a key target for immunotherapy in cancer patients. The major components of the tumor microenvironment include tumor-associated macrophages, type 2 natural killer T cells, regulatory T cells, and myeloid-derived suppressor cells (MDSCs)^3^. MDSCs play pivotal effects in multiple steps of tumorigenesis and metastasis^3^. MDSCs are derived from bone marrow stem cells. MDSCs are a heterogeneous population of cells that interact with T cells, dendritic cells, macrophages and natural killer cells. MDSCs have strong immunosuppressive activities. The detection of MDSCs in cancer specimens has been associated with a poor patient prognosis and resistance to cancer therapies 4,5. The higher the number of MDSCs in patients with late-stage III or IV gastric cancer, the worse the prognosis 6. A better understanding of the immunosuppressive cells of gastric cancer will allow for the appropriate treatment and for future drug development.

Serine/threonine-protein kinase 24 is a subfamily of the germinal center kinase-III (GCK-III) family and is encoded by the *STK24* gene in humans. STK24 is also known as Mammalian STE20-like protein kinase 3 (MST-3)^7^. In previous studies, the roles of STK24/MST3 have been implicated in the control of cancer cell migration and the regulation of neutrophil degranulation 8-10. The functions of GCKs are involved in inflammatory responses and participate in cancer and immunological disorders 11. The expression of STK24/MST3 in the stomach has been observed in normal, intestinal metaplasia and in portions of tumors 12. The immunological effects of STK24 in gastric cancer are less well understood. The current study explores the role of STK24 in tumorigenesis and the immune response of an orthotopic animal model of gastric cancer.

## Materials and Methods

### Reagents and antibodies

N-nitro-N-methylurea (MNU) was purchased from Sigma-Aldrich (St. Louis, MO). The following antibodies (Abs) were used in this study and were purchased from BD PharMingen (San Diego, CA): mouse anti-CD4 PE (H129.19), anti-CD8a PE (53-6.7); anti-CD11b PE (M1/70), anti-F4/80 PE (BM8), anti-Ly6G FITC (1A8), anti-Ly6C FITC (AL-21) mAb. The anti-CD44 PE (IM7), PE rat IgG1 and FITC rat IgG2a isotype control Abs were purchased from eBioscience. The following antibodies were used in this study: mouse anti-ASS1 (BD Transduction Laboratories, San Jose, CA, USA); anti-MST3 (EP1468Y) (Abcam, United Kingdom); mouse anti-JAK1 (BD Biosciences, San Jose, CA); rabbit anti-STAT3, rabbit anti-CCND1, rabbit anti-AKT1 and peroxidase-conjugated goat anti-rabbit IgG (Cell Signaling, Boston, MA, USA); mouse anti-β-actin (GeneTex, Inc., San Antonio, TX, USA); and peroxidase-conjugated sheep anti-mouse IgG (Chemica, San Diego, CA, USA).

### Ethics statement

MNU-induced gastric tumors were generated in male mice as previously reported 13. P53 knockout mice were a kind gift from Dr. CL Wu (National Cheng Kung University, Tainan, Taiwan). To genotype each mouse, DNA samples were extracted from tail samples using a *QIAamp DNA Mini Kit* (Qiagen, Valencia, CA) as previously described 13. Six-week-old NOD/SCID mice were purchased from the Laboratory Animal Center of National Cheng Kung University (Tainan, Taiwan) and were maintained under pathogen-free conditions. The methods were carried out in accordance with the approved guidelines.

### MNU-induced gastric carcinoma and the establishment of a mouse gastric carcinoma cell line

**Figure 1:**
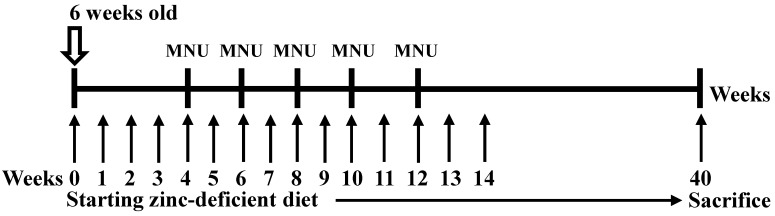
** Experimental design used to induce gastric cancer in p53 knockout (-/-) mice.** Six-week-old p53 KO male mice were given drinking water that contained 30 ppm MNU ad libitum for 5 weeks and were fed a zinc-deficient diet that contained 1 ppm zinc ad libitum. The mice were sacrificed after 40 weeks, and the established tumors were excised and cultured.

Custom-formulated, egg white-based zinc deficient diets contained 1 ppm zinc (TestDiet). MNU was dissolved in distilled water and was freshly prepared three times per week. Six-week-old p53 knockout mice were fed a zinc-deficient (ZD) diet ad libitum and were given drinking water that was treated with 30 p.p.m. MNU 1 week on and 1 week off; then, the mice were sacrificed after 40 weeks 13,14 (Figure 1). The tumors were then separated from the stomach tissue as previously described 15. The tumor cells were orthotopically implanted into syngeneic mice after 7 cycles of stepwise selection, and the resultant tumors were isolated and cultured in vitro to establish the M12 cell line. A highly tumorigenic and metastatic cell line was established, and we orthotopically implanted tumor cells into syngeneic C57BL/6 mice. M12 cells were maintained in high-glucose DMEM supplemented with 10% FBS (Gibco, Life Technologies, Grand Island, NY, USA), 100 U/mL penicillin, and 100 µg/mL streptomycin. The M12 cell line was subcultured for more than 3 years without any apparent phenotypic changes. In the current study, the M12 cell line established by ourselves has been deposited under the Food Industry Research and Development Institute in Taiwan (accession number BCRC960512). A highly tumorigenic stomach cancer cell line (3IB2) was maintained in high-glucose DMEM supplemented with 10% FBS (Gibco, Life Technologies, Grand Island, NY, USA), 100 U/mL penicillin, and 100 µg/mL streptomycin 16. AGS human gastric cancer cells were obtained from the Bioresource Collection and Research Center (BCRC, Food Industry Research and Development Institute, Hsinchu, Taiwan), and MKN45 cells were kindly provided by Dr. MD Lai (National Cheng Kung University, Tainan, Taiwan). The AGS and MKN45 cell lines were maintained in RPMI 1640 medium containing 10% fetal bovine serum (FBS) and 1% penicillin/streptomycin. M12 cells were examined by a Mycoplasma PCR Detection Kit according to the manufacturers' instructions (Applied Biological Materials) and no signs of mycoplasma infection was detected. The AGS and MKN45 cell lines were authenticated by DNA (short-tandem repeat) profiling at the Bioresource Collection and Research Center in 2013.

### Orthotopic and experimental metastatic model of gastric cancer

The mice were anesthetized by an intraperitoneal injection of Zoletil (50 mg/kg; Parnell Laboratories, Alexandria, NSW, Australia) and xylazine (10 mg/kg; Troy Laboratories, Glendenning, NSW, Australia). Then, a small incision over upper midline of abdomen was made in the mice under anesthesia, and stomach serosa were exteriorized. Total 1x10^6^ tumor cells in 0.05 ml of PBS were injected into the subserosal layer of the greater curvature of the gastric wall using a 1-cc U-100 disposable insulin syringe (Becton-Dickinson, Franklin Lakes, NJ, USA). To characterize the tumorigenic properties of the M12 cell line *in vivo*, we evaluated the tumor growth in the syngeneic mice by the orthotopic transplantation of tumor cells directly into the stomach serosa as previously described 15. The metastatic abilities of the M12 cell clones *in vivo* were evaluated using a hepatic metastasis model 16, and 1x10^6^ tumor cells in 0.05 mL of PBS were injected intrasplenically as previously described 16. After the growth of hepatic metastasis, the C57BL/6 mice were sacrificed, and the hepatic tumors were examined macroscopically and microscopically. Formalin-fixed, paraffin-embedded sections of the stomach, liver and spleen were used for hematoxylin and eosin (H&E) staining. Each animal experiment was performed at least twice.

### CRISPR knockout *STK24* plasmid construction, transfection, and stable cell line generation

sgRNAs targeting mouse *STK24* and human *STK24* were purchased from the National RNAi Core Facility (Academia Sinica, Taiwan; http://rnai. genmed.sinica.edu.tw). The DNA sequences for generating mouse sg*STK24*-RNA1 and sg*STK24*- RNA2 were as follows: sg*STK24*-RNA1 forward, 5'- CACCG CTGGG TATCC GTCAG CTGGC-3' and sg*STK24*-RNA1 reverse, 5'- AAACG CCAGC TGACG GATAC CCAGC-3'; sg*STK24*-RNA2 forward, 5'- CACCG GTCGA TAAGC TCGGT CAAGT-3' and sg*STK24*-RNA2 reverse, 5'- AAACA CTTGA CCGAG CTTAT CGACC-3'. For the control plasmid, the pEGFP sequence was inserted into the construct. A 20-bp guide sequence targeting the *STK24* DNA was selected from a database of predicted high-specificity protospacer adjacent motif (PAM) target sites in the mouse and human exome. Two complementary oligos containing the *STK24* guide sequence and BsmBI (NEB, Beverly, Ma) ligation adapters were synthesized. Each oligo was phosphorylated and annealed using T4 Polynucleotide Kinase (New England Biolabs, Ipswich, MA, USA). The annealed oligo was ligated into the BsmBI-digested- pU6-sgRNA.pPuro (gRNA). The sequence of the construct was verified by DNA sequencing. M12 cells were seeded into 24-well plates until they reached 70-80% confluence. The Cas9/gRNA vector construct was introduced into the cells by transfection with Lipofectamine 3000 (Invitrogen) for 48 h. To create a stable cell line, the selection was performed with puromycin (1 μg/ml) (Sigma-Aldrich) for 2 weeks. Single cell clones of the transfectants were selected using the limiting dilution method. To monitor the efficacy of *STK24* silencing, STK24 expression in the stable transfectants was analyzed by western blotting.

### Semi-quantitative reversed transcription- polymerase chain reaction (RT-PCR)

Total RNA was extracted from 1×10^6^ tumor cells, purified using the RNeasy Kit according to the manufacturer's instructions (Qiagen, Valencia, CA), and converted to cDNA by Moloney Murine Leukemia Virus (M-MLV) reverse transcriptase with oligo (dT) primer in the presence of RNAsin (Promega, Madison, WI). Semi-quantitative PCR for p53 wild-type, p53 mutant, MUC1, MUC5AC, COX2, c-myc, c-met, MMP-2, MMP-9 and β-actin were performed as described previously^13^,^15^. Primers used in RT-PCR were as follows: mouse cyclin A2 forward, 5'- AGAGGCAGCCAGACATCACT-3'; mouse cyclin A2 reverse, 5'- AGCCAAGTCAAAAGCAAGGA-3'; mouse p21 forward, 5'- GTACTTCCTCTGCCCTGCTG -3'; mouse p21 reverse, 5'- ACACTATCCTGGGCATTTCG-3'; mouse ASS1 forward, 5'- AGAGCCCCCTGGAGTATGGAT-3'; mouse ASS1 reverse, 5'- ATGAGCGTGGTAAAGGATGG-3'; human ASS1 forward, 5'-CAGACGCTATGTCCAGCAAA-3'; human ASS1 reverse, 5'-TGCTTTGCGTACTCCATCAG-3'.

### Western blot analysis

The tumor tissues and tumor cell lysates were prepared and analyzed by SDS-PAGE as previously described 16. The tumor cells were washed twice with PBS and were lysed with ice-cold RIPA buffer. The RIPA buffer (20 mM Tris-HCl pH 7.5, 1 mM EDTA, 150 mM NaCl, 1% NP-40 and 1% SDS) contained one protease inhibitor tablet (cOmplete, Mini, EDTA-free Protease Inhibitor Cocktail, Roche). The pEGFP control (EGFP-Ctrl) and sgSTK24-expressing cells were removed with a cell scraper and were collected in a tube for 15 minutes at 4°C. The samples were centrifuged for 10 minutes at 13,000 RPM at 4°C, and the supernatant was transferred to an Eppendorf tube. The total proteins of the cell lysates were assayed quantitatively using the Bio-Rad Bradford assay (Mississauga, ON). Cell lysates were immunoblotted using anti-STK24, anti-AKT1, anti-ASS1, anti-JAK1, anti-CCND1, anti-STAT3 and anti-β-actin antibodies. Immunodetection was performed using HRP-based SuperSignal Chemiluminescent Substrate (Pierce, Rockford, IL, USA). For the quantification, the bands were measured using an AlphaImager 2200 system (Alpha Innotech) and were normalized to the band density of β-actin.

### Patients

Fresh tissue specimens were collected from 39 patients with gastric adenocarcinoma who underwent radical resection at National Cheng Kung University Hospital (Tainan, Taiwan) between August 2003 and August 2008. The mean age was 63±13 years old (range 35-87 years old; 25 males, 14 females). Only patients with adenocarcinomas were selected and the cancer stage were defined as 7 (18%) stage I, 13 stage II (33%), 15 stage III (40%) and 4 stage IV (10%) according to AJCC TNM Stage (American Joint Committee on Cancer Tumor Node Metastasis stage). Total 39 pairs of cancerous tissues and matched, adjacent normal gastric mucosa were collected and analyzed as previously described (30). The specimens were preserved in the Human Biobank within the Research Center of Clinical Medicine of the National Cheng Kung University Hospital (Tainan, Taiwan). The present study was approved by the Institutional Review Board of the National Cheng Kung University Hospital (NCKUH IRB no. ER-98-259).

### Cell proliferation assay

M12 (2 x 10^3^ cells/well) cells were seeded in triplicate into 96-well plates and were incubated at 37°C in 5% CO2. The number of viable cells was measured at daily intervals (24, 48, and 72 h) with the CellTiter 96 AQueous One Solution Cell Proliferation Assay (Promega, Madison, WI, USA) according to the manufacturer's instructions. Then, 20 μL of CellTiter One Solution was added to each well. After a 1 h incubation, the absorbance was measured at 490 nm with an ELISA plate reader.

### Colony formation assay

The pEGFP control (EGFP-Ctrl) and sgSTK24-expressing cells were seeded at a density of 500 cells/ well in six-well culture plates and were cultured at 37°C for 10 days. The cells were fixed with methanol/ acetic acid (3:1) and were stained with a 0.5% crystal violet solution, washed with PBS, and photographed.

### Flow cytometry analysis

To characterize CD44 expression in M12 cells, 1×10^6^ cells were stained with anti-CD44 conjugated with phycoerythrin antibody and evaluated on a flow cytometer (FACScan, Becton Dickinson, CA)^15^. To characterize the immune cells of the spleen in the tumor-bearing mice *in vivo*, the spleen was isolated and subjected to flow cytometry as previously described 16,17.

### Cytokine array

To collect the culture supernatants, the control-pEGFP and sgSTK24-1.2 cells were cultured for 48 h in serum-free medium, after which, the supernatants were collected and measured using a Mouse Cytokine Antibody Array III Kit (RayBiotech) according to the manufacturer's instructions.

### Bioinformatics and statistical analyses

The collected gene expression data from the Gene Expression Omnibus (GEO) database (http://www.ncbi.nlm.nih.gov/geo/) is under the series accession number GSE15459. From the GSE15459 dataset, the data of 200 gastric cancer patients were collected. Raw data correction and normalization were performed with the Robust Multichip Average (RMA). The Robust Multichip Average (RMA) signal was computed for gene-level probeset summaries with Affymetrix Expression Console Version 1.3.1.187 (http://www.affymetrix.com). The genes that had low expression levels across all 200 tumor samples included in the study were ranked from the lowest expression level to the highest expression level across all the samples with the GENE-E package as previously described 18. The difference between the average expression in invasive gastric cancer patients and the average expression in other subtype samples was computed; in addition, the fold-change between the two sample groups was also computed. A search of the Oncomine database (http://www.oncomine.com) was initially conducted to systematically assess the expression levels of the *STK24* gene in gastric cancer as previously described 19,20. The prognostic value of the *STK24* gene in gastric cancer was also analyzed using the Kaplan-Meier Plotter (http://kmplot.com/analysis/) as described previously 20,21. The *STK24* gene probe sets were available and are listed as 208855_s_at; then, the patients were split into two groups according to the median expression or according to the expression at the best cut-off value. GraphPad Prism (version 4.00 for Windows; GraphPad Software, San Diego, CA, USA; www.graphpad.com) was used for the analyses. The data are expressed as the mean ± standard deviation (s.d.). Statistical analyses were performed using Student's *t*-test or one-way ANOVA followed by Tukey's test. Statistical analyses between the two groups were performed using Student's *t*-test. One-way ANOVA was used for multiple group comparisons. The protein interactions were downloaded from STRING version 8.0 on April 2, 2019 (http://string.embl.de).

## Results

### Establishment of an orthotopic transplantable model of gastric cancer in immunocompetent C57BL/6 mice

**Figure 2:**
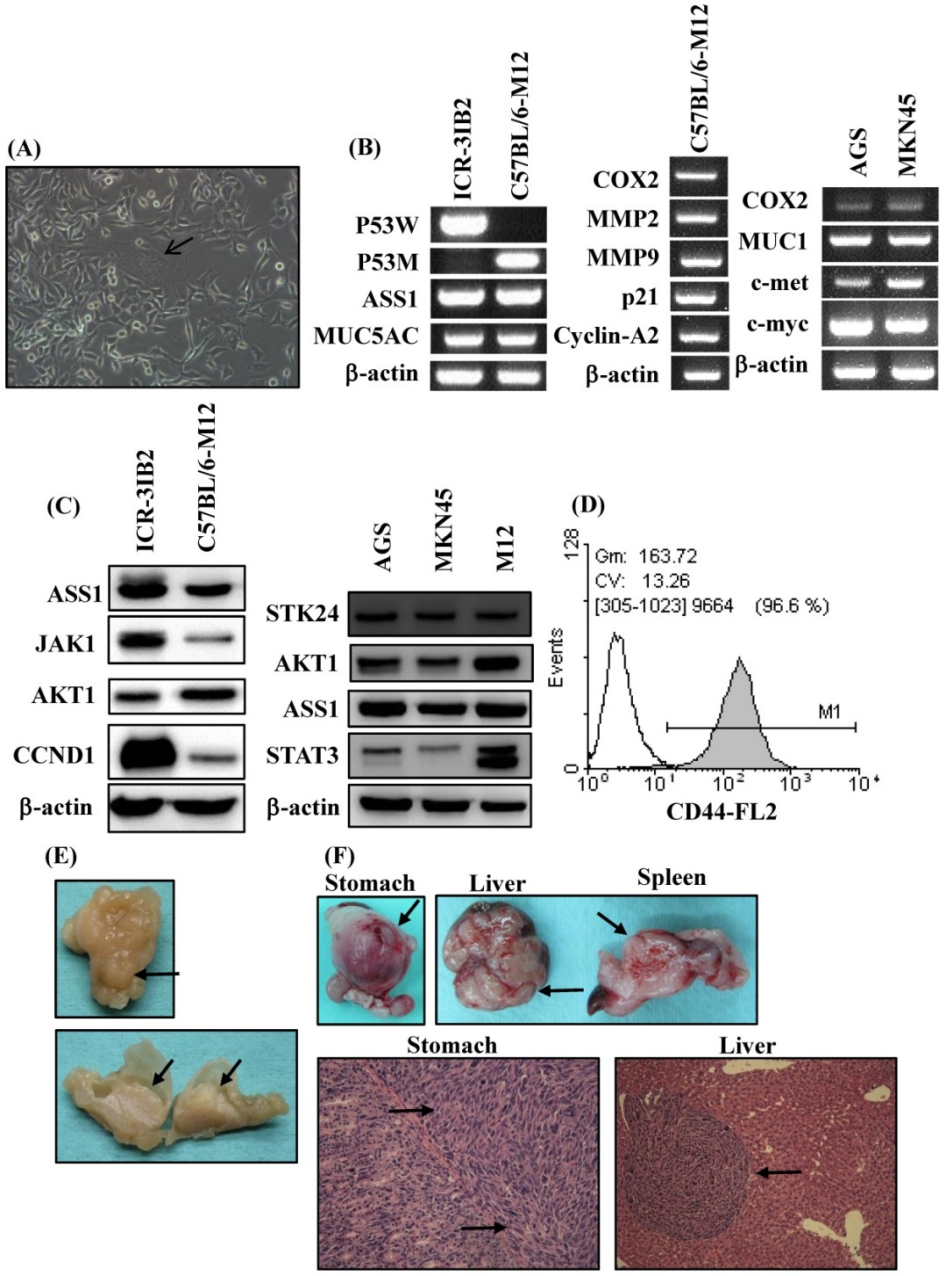
** Phenotypic characteristics of the mouse gastric carcinoma cell line M12 *in vitro* and *in vivo*.** (A) The morphology was observed at a high confluence under an optical microscope. The original magnification was 200x. M12 cells exhibited an epithelial‐like morphology. Giant cell formation illustrating enlarged and flattened morphology was indicated by arrows. (B) The expression levels of gastric carcinoma‐related genes in the mouse gastric carcinoma cell lines were determined by RT‐PCR. (C) The ASS1, JAK1, AKT1, CCND1, STK24 and STAT3 protein expression levels were determined by western blot analyses. STAT3 isoform expression appears as STAT3α (86 kDa) and STAT3β (79 kDa). (D) The expression of the cell surface marker CD44 was determined by flow cytometry. (E) Macroscopic views of orthotopic stomach tumor. A protruding type of stomach cancer was developed in a tumor-bearing C57BL/6 mouse model. (F) The tumorigenicity and metastasis potentials of M12 cells with different routes of implantation are shown. The macroscopic appearance of the tumor masses after orthotopic implantation and after the intrasplenic injection of M12 cells are shown. The histology of the orthotopic stomach tumor and liver metastases on day 20 are shown. The tumor cells are indicated by arrows.

In the current study, an orthotopic animal model of gastric cancer was developed in syngeneic inbred C57BL/6 mice. Inbred C57BL/6 mice are able to provide a stable genetic background for mutants and allow for genetic alteration, which is a very useful approach for tumor immunology studies. The adherent monolayer growth and the spindle‐shaped morphology of the M12 cell line was established from the gastric carcinoma induced in MNU‐treated C57BL/6 mice (Figure 2A). M12 cells tended to form multinuclear giant cells (Figure 2A). To evaluate the similarity of M12 cells to human gastric cancer, we analyzed the molecular and biochemical characteristics, and gross appearance of orthotopic gastric cancer from M12 cells. In a previous study, we established a mouse 3IB2 gastric cancer cell line from outbred ICR mice 16. M12 cells retained the expression of ASS1 and MUC5AC as was determined by RT‐PCR (Figure 2B, left). PCR was used for the genotyping analysis of mouse p53 and revealed that the 3IB2 cells were positive for wild-type p53 and that the M12 cells contained mutated p53. To evaluate the similarity of M12 cells to human gastric cancer cell lines-AGS and MKN45 cells, we analyzed the gene expression profile of this mouse gastric cancer cell line. M12 cells retained the expression of COX2, MMP2, MMP9, p21 and cyclin A2 as determined by RT-PCR (Figure 2B, middle). The expression of several gastric carcinoma-associated genes was also measured with RT-PCR in AGS and MKN45 cells. Increased expression of COX2, MUC1, c-met and c-myc was observed in AGS and MKN45 cells (Figure 2B, right).Western blot analysis revealed that the 3IB2 and M12 cells were positive for the following proteins: argininosuccinate synthetase 1 (ASS1), janus kinase 1 (JAK1), AKT1, and cyclin D1 (CCND1) (Figure 2C, left). Western blot analysis revealed that AGS, MKN45 and M12 cells were positive for STK24, AKT1, ASS1 and signal transducer and activator of transcription 3 (STAT3) (Figure 2C, right). Then, we analyzed the expression of the potential stem cell marker CD44 in M12 cells. A flow cytometry analysis revealed the presence of CD44 (Figure 2D). To characterize the tumorigenic and metastatic properties of the M12 cell line *in vivo*, we evaluated the tumor growth and metastasis in C57BL/6 syngeneic mice by the two following implantation routes: the orthotopic injection of tumor cells directly into the stomach and the intrasplenic injection of tumor cells. After the orthotopic transplantation of M12 cells directly into the serosa layer of the stomach, thickening of the gastric wall and tumor growth in the stomach were detected for each mouse. The gross appearance of the stomach exhibiting a protruded pattern (Figure 2E). The M12 cells showed tumor growth in the stomach with an incidence of 100% and retained the characteristics of histopathologically poorly differentiated carcinoma on day 20 (Figure 2F). The M12 cells then metastasized to the liver with an incidence of 100% when the cells were injected into the spleen on day 20 (Figure 2F).

### Association of* STK24* gene expression with gastric cancer patient survival

**Figure 3:**
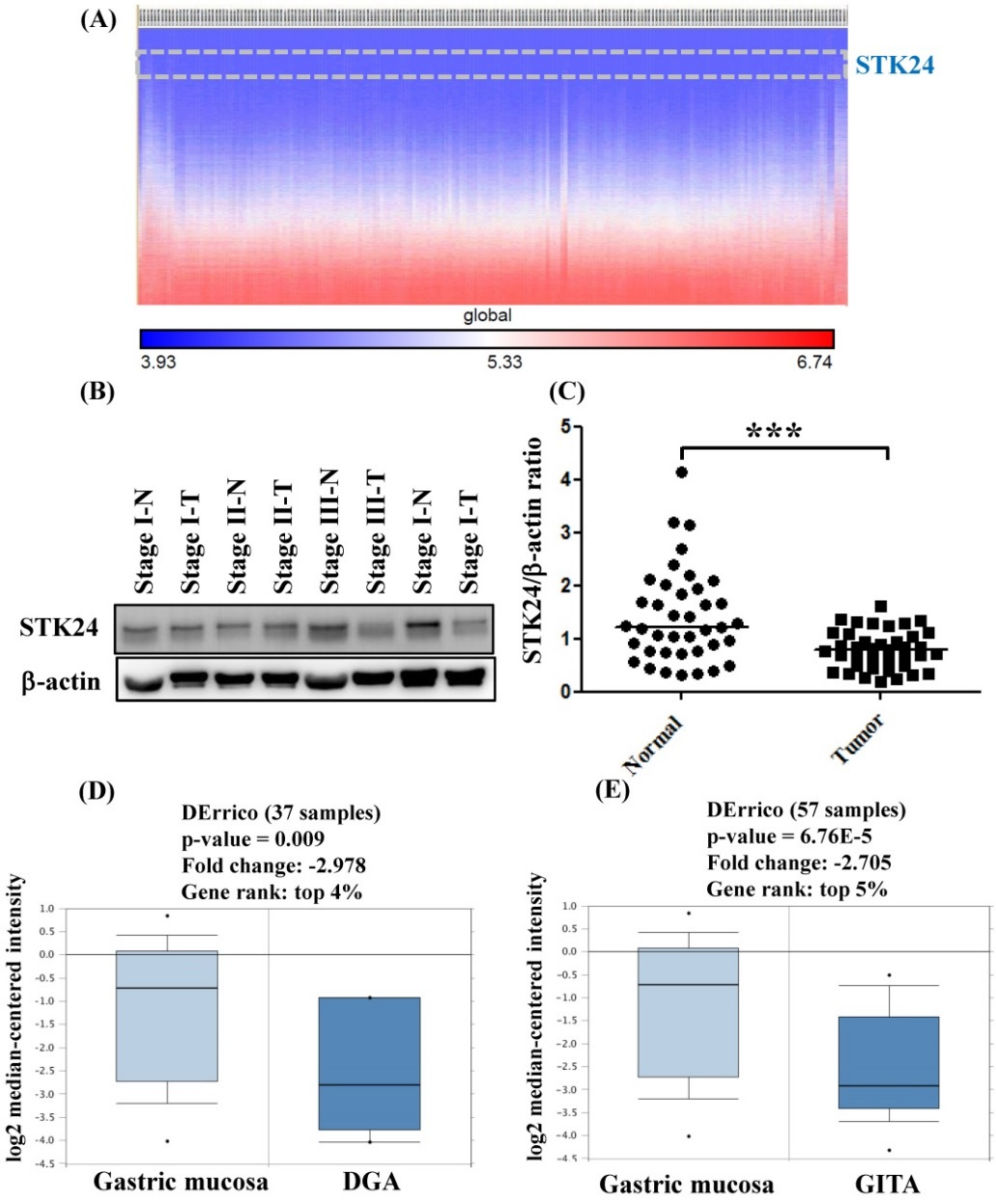
** Gene expression of *STK24* in gastric cancer from the Oncomine database.** (A) The heatmap shows genes ranked from the lowest levels of expression to the highest levels of expression for raw expression values across 200 gastric cancer patients. The lowest expression for STK24 (inset, top panel) ranked 4351 out of over 34180 genes in the whole microarray dataset, which also demonstrates that STK24 had low expression in the top 12.7% of genes from these gastric cancer patients. (B) STK24 expression was measured in specimens of gastric cancer and normal stomach tissues by western blot analysis. (C) STK24 expression is presented relative to that of β-actin (STK24/β-actin ratio). The STK24/β-actin ratio of 39 samples was measured in specimens of normal stomach tissues (N) and gastric cancer tissues (T). The expression patterns of *STK24* in (D) diffuse gastric adenocarcinoma (DGA) and (E) gastric intestinal-type adenocarcinoma (GITA) of gastric cancer datasets were obtained from the Oncomine database. ***P < 0.0001.

**Figure 4:**
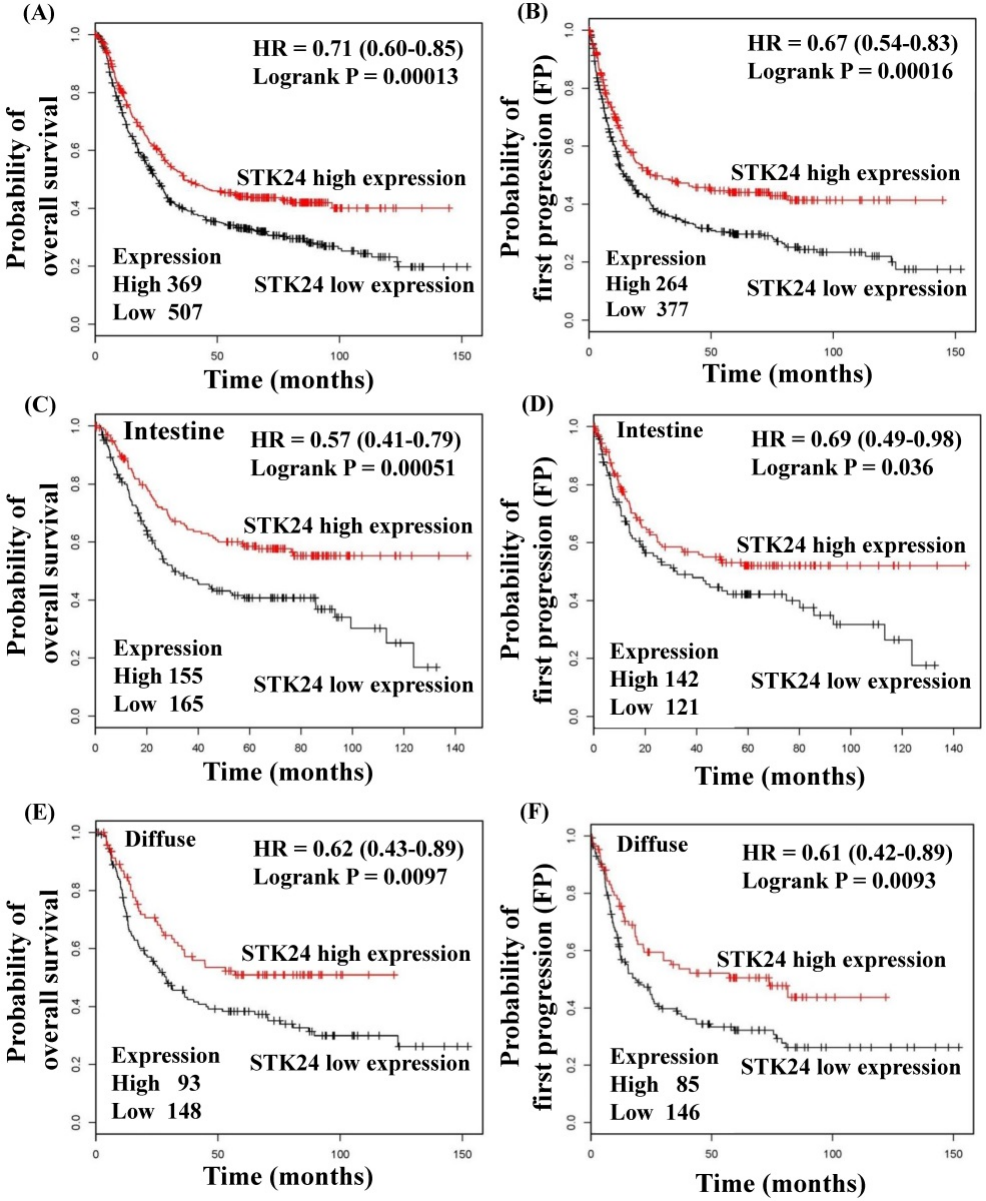
** Kaplan‑Meier survival curves regarding *STK24* gene expression in patients with gastric cancer.** The low expression of *STK24* was associated with a lower OS (A) and PFS (B). The low expression of *STK24* was correlated with a low OS (C) and PFS (D) in different intestinal types. The low expression of *STK24* was correlated with lower OS (E) and PFS (F) **in** diffuse types. The total number of patients in the low‑and high‑expression groups, as well as the HR and P‑values (log‑rank), are included. HR, hazard ratio; OS, overall survival; PFS, progression‑free survival.

To explore whether STK24 plays an important role in gastric cancer development, we further analyzed a published microarray dataset (GSE15459) and identified that these 200 gastric cancer patients had low expression levels of STK24. These data demonstrate that the expression level of STK24 ranked 4351 out of the expression levels of the 34180 genes in the whole microarray dataset. This result indicates that the expression of the *STK24* gene is in the top 12.7% of the genes that are downregulated in gastric cancer tissue (**Fig. 3A**). These data indicate that STK24 may play an important role in gastric cancer development in patients. The expression levels of the STK24/MST3 protein were examined by a western blot analysis in the tumor and adjacent normal gastric tissues of 39 patients (**Fig. 3B**). An analysis of the relative expression of the STK24 protein in the normal tissues and gastric cancer tissues indicated that the STK24/β-actin ratio in the normal samples was significantly greater than the ratio in the gastric cancer samples (P<0.0001) (**Fig. 3C)**. The data for *STK24* transcript expression were extracted from the Oncomine database for gastric cancer, and the focus was on normal vs. cancer patient datasets. *STK24* expression was significantly decreased in diffuse gastric adenocarcinoma (DGA) (**Fig. 3D)** and in gastric intestinal-type adenocarcinoma (GITA) (**Fig. 3E**)^22^. To determine the clinical significance of STK24 in human gastric cancer, we analyzed the expression of the *STK24* gene in the Kaplan‑Meier Plotter and Oncomine databases. The high expression of the* STK24* gene was correlated with better overall survival (OS) and first progression (FP) in the Kaplan-Meier survival curves (**Fig. 4, A and B**). According to the Lauren classification, gastric cancer is classified as intestinal and diffuse. The high expression of the *STK24* gene was correlated with a better overall survival (OS) and first progression (FP) in both the intestinal (**Fig. 4, C-D**) and diffuse types (**Fig. 4, E-F**). Thus, the bioinformatic analyses using the Kaplan‑Meier Plotter and Oncomine databases indicate that *STK24* expression was involved in cancer progression. These results suggest that the downregulation of the *STK24* gene was associated with the poor prognosis of gastric cancer patients.

### Suppression of STK24 expression did not influence cell proliferation in M12 gastric cancer cells

**Figure 5:**
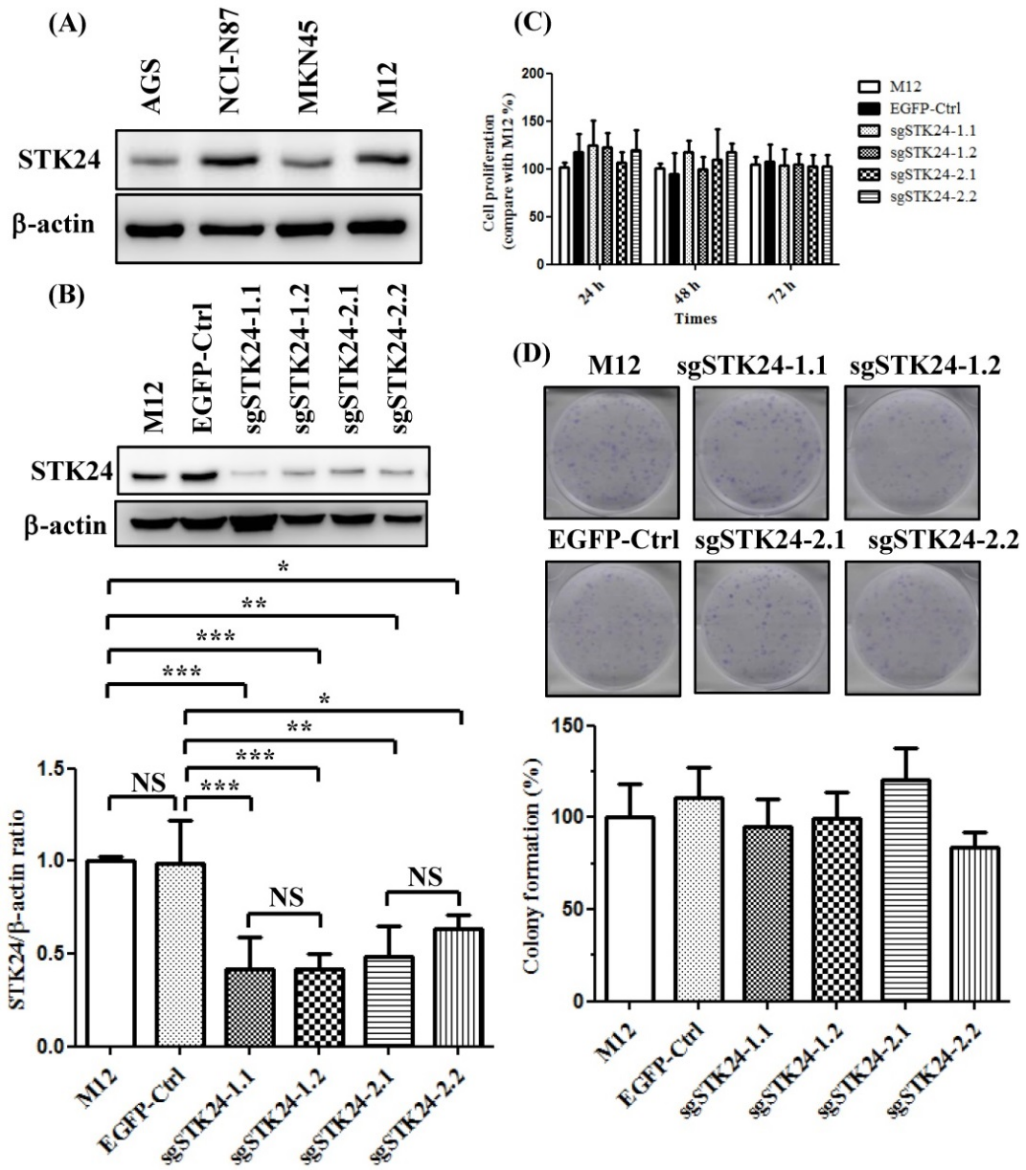
** STK24 silencing in mouse M12 cells did not influence the cell proliferation rates.** (A) The protein expression of STK24 was determined in AGS, NCI-N87, MKN45 and M12 cells. (B) STK24 protein expression was determined in mouse M12 cells and in STK24 sgRNA stable transfectants. The results of the western blot analyses of protein expression were obtained from three independent experiments. The bars represent the mean ± the SD. P, parental cells; EGFP-Ctrl, EGFP control; sgSTK24-1.1, sgSTK24-1.2, sgSTK24-2.1 and sgSTK24-2.2, STK24-specific sgRNAs 1 and 2. (C) The proliferation rates of M12 cells and STK24 sgRNA stable transfectants were determined at 24, 48, and 72 h. (D) Representative images of the colony formation assays with the EGFP-Ctrl and sgSTK24-expressing cell clones. A quantitative analysis of the colony numbers is shown in the bottom panel. The data are expressed as percentages relative to the number of M12 cells and are representative of two different experiments. NS, not significant; *P < 0.01; **P < 0.001; ***P < 0.0001.

We first analyzed the constitutive protein expression of STK24 in human AGS, NCI-N87, MKN45, and mouse M12 gastric cancer cell lines (Fig. 5A). We knocked down the expression of the *STK24* gene using two different sgRNAs. We established stable cell lines with control pEGFP vectors and then transduced them with the following gRNA constructs of* STK24*: sgSTK24-1.1, sgSTK24-1.2, sgSTK24-2.1, and sgSTK24-2.2. The successful suppression of the STK24 protein in M12 cancer cells was demonstrated by western blotting (Fig. 5B). The cell proliferation rates (Fig. 5C) and colony formation rates (Fig. 5D) of the pEGFP control (EGFP-Ctrl) and sgSTK24-expressing cells were similar. Therefore, the suppression of STK24 did not affect the cell growth rates of the mouse M12 cancer cell lines.

### Suppression of STK24 promoted the tumor growth of M12 cells *in vivo*

**Figure 6:**
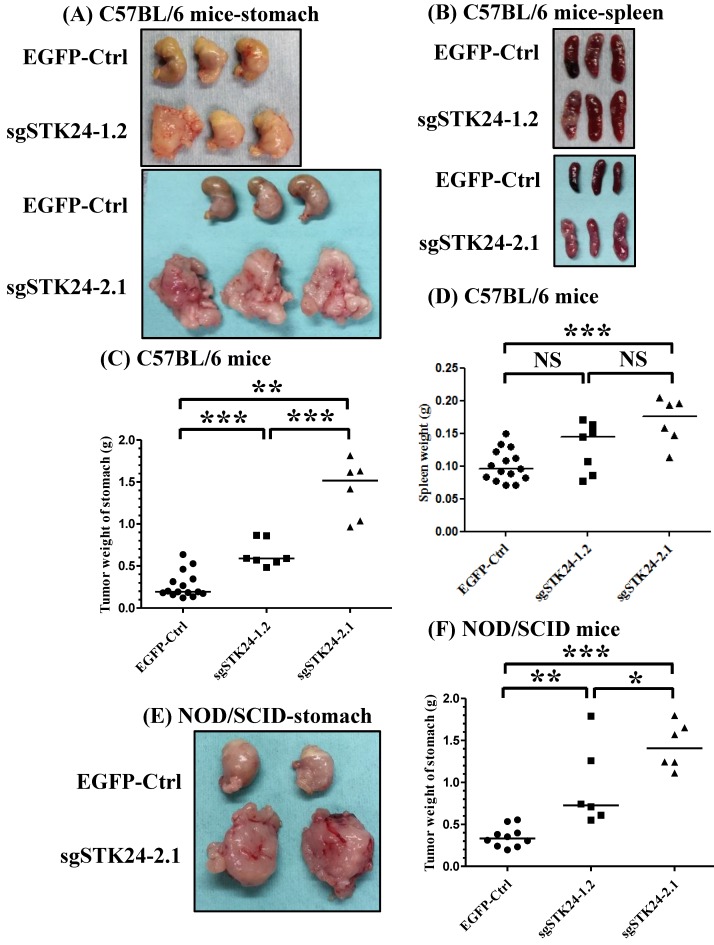
** STK24 silencing in M12 cell clones enhances tumor growth *in vivo*.** The macroscopic appearance of the tumor masses (A) and spleen (B) after the orthotopic injection of either the pEGFP-control (EGFP-Ctrl) or the sgSTK24-expressing cell clones of tumor-bearing C57BL/6 mice on Day 16 are shown. The stomach weights (C) and spleen weights (D) of the EGFP-Ctrl, sgSTK24-1.2 and sgSTK24-2.1 tumor-bearing C57BL/6 mice on Day 16 are shown. The macroscopic appearances of the tumor masses (E) and the stomach weights (F) of EGFP-Ctrl, sgSTK24-1.2 and sgSTK24-2.1 tumor-bearing NOD/SCID mice on Day 16 are shown. *P < 0.01; **P < 0.001; ***P < 0.0001. The results are expressed as the mean stomach weight and the mean spleen weight, and the data are averaged from two independent experiments.

To investigate the role of STK24 in a native tumor environment, we examined the effects of STK24 knockdown in an orthotopic immune-competent animal model. One million pEGFP control (EGFP-Ctrl) or sgSTK24-expressing cells were implanted orthotopically in C57BL/6 mice, and macroscopic tumor nodules, which are indicative of tumor formation, were detected (Fig. 6A-B). The tumor weights in the mice injected with EGFP-Ctrl cells were significantly lower than the tumor weights of the mice injected with the sgSTK24-1.2 (Fig. 6C) and sgSTK24-2.1 cells (Fig. 6D) on day 16 after the injections. These results demonstrate that the knockdown of STK24 promoted the tumor growth of gastric cancer cells *in vivo*; this suggests that STK24 plays an important role in the tumorigenesis of gastric cancer.

Since the growth of the sgSTK24-expressing cells was not altered *in vitro*, we hypothesized that the *in vivo* changes in tumor growth may result from altered tumor-microenvironment interactions. To assess the potential immunological effects of STK24 on tumor progression, the tumor growth was measured in nonobese diabetic/severe combined immunodeficient (NOD/SCID) mice. The NOD/SCID mice lacked NK cells, B and T cells; however, these mice did have monocytes, neutrophils, and myeloid-derived mononuclear cells. One million pEGFP control (EGFP-Ctrl) or sgSTK24-expressing cells were implanted orthotopically into immunodeficient NOD/SCID mice, and the macroscopic tumor nodules indicative of tumor formation were detected (Fig. 6E-F). In the absence of NK cells, B cells and T cells, the tumor weights of the mice injected with EGFP-Ctrl cells were significantly lower than the tumor weights of the mice injected with the sgSTK24-1.2 (Fig. 6F) and sgSTK24-2.1 cells (Fig. 6F) on day 16 after the injection. The results indicate that the effects of STK24 were dependent on the presence of myeloid-derived suppressor cells.

### The frequency of CD11b^+^Ly6C^+^ MDSCs and F4/80^+^ macrophages is increased in the spleens of tumor-bearing mice

**Figure 7:**
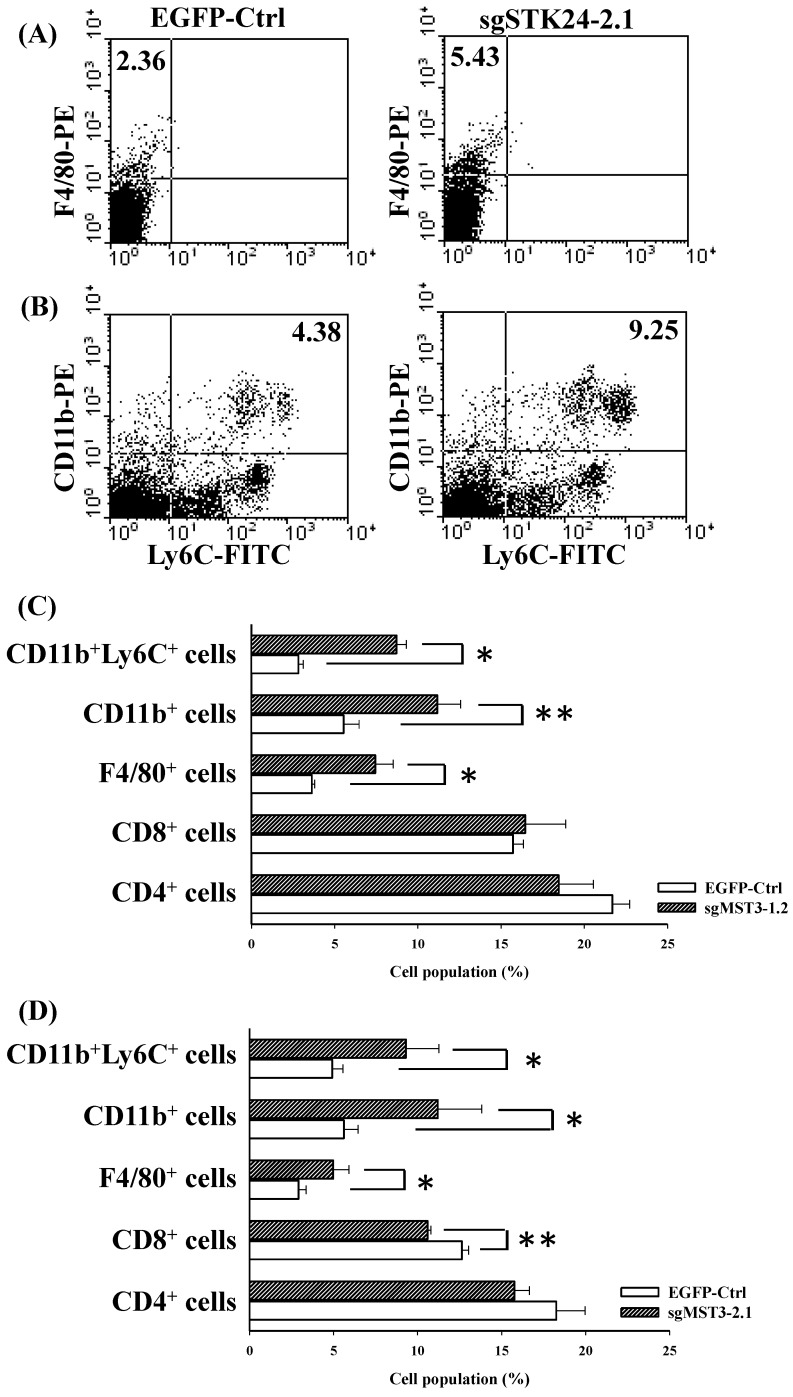
** The frequency of F4/80^+^ macrophages and CD11b^+^Ly6C^+^ cells was significantly increased in the spleens of STK24-silenced tumors.** Flow cytometry was performed on the spleens of EGFP-Ctrl, sgSTK24-1.2 and sgSTK24-2.1 mice after M12 tumor cell implantation on Day 16. (A) F4/80^+^ macrophages and (B) CD11b^+^Ly6C^+^ cells were isolated from the spleens of EGFP-Ctrl and sgSTK24-2.1 mice. The numbers shown are the percentage of total cells. (C) The cell populations of CD4 T cells, CD8 T cells, F4/80^+^ macrophages, CD11b^+^ cells, and CD11b^+^Ly6C^+^ cells of splenocytes were analyzed by flow cytometry 16 days after EGFP-Ctrl and sgSTK24-1.2 tumor cell implantation. (D) The cell populations of CD4 T cells, CD8 T cells, F4/80^+^ macrophages, CD11b^+^ cells, and CD11b^+^Ly6C^+^ cells of splenocytes were analyzed by flow cytometry 16 days after EGFP-Ctrl and sgSTK24-2.1 tumor cell implantation. *P < 0.05; **P < 0.01. The results are expressed as the mean cell population ± SD, and the data are averaged from two independent experiments.

To study STK24-mediated immunity in gastric cancer, we investigated the splenocyte subtypes in an orthotopic animal model of gastric cancer. The frequency of F4/80^+^ macrophages was significantly increased in the spleens of sgSTK24-1.2-bearing and sgSTK24-2.1-bearing mice (Fig. 7A, C-D). MDSCs are characterized to have either the CD11b^+^Ly6C^+^ or the CD11b^+^Ly6G^+^ phenotype. The CD11b^+^Ly6C^+^ subtype was increased significantly in the spleens of sgSTK24-1.2-bearing and sgSTK24-2.1-bearing mice (Fig. 7B, C-D). The CD11b^+^Ly6G^+^ subpopulation was not expanded. However, no significant difference was observed in the total number of CD4^+^ T cells in the spleens of sgSTK24-1.2 and sgSTK24-2.1-bearing mice (Fig. 7C-D). Then, the frequency of the CD8^+^ T cells of splenocytes significantly increased in the sgSTK24-2.1-bearing mice (Fig. 7D) but did not increase in the sgSTK24-1.2-bearing mice (Fig. 7C). These results indicate that the silencing of STK24 in tumors induced the expansion of CD11b^+^Ly6C^+^ cells and F4/80^+^ macrophages cells* in vivo*.

### Suppression of STK24 increases cytokine/ chemokine secretion by M12 gastric cancer cells

**Figure 8:**
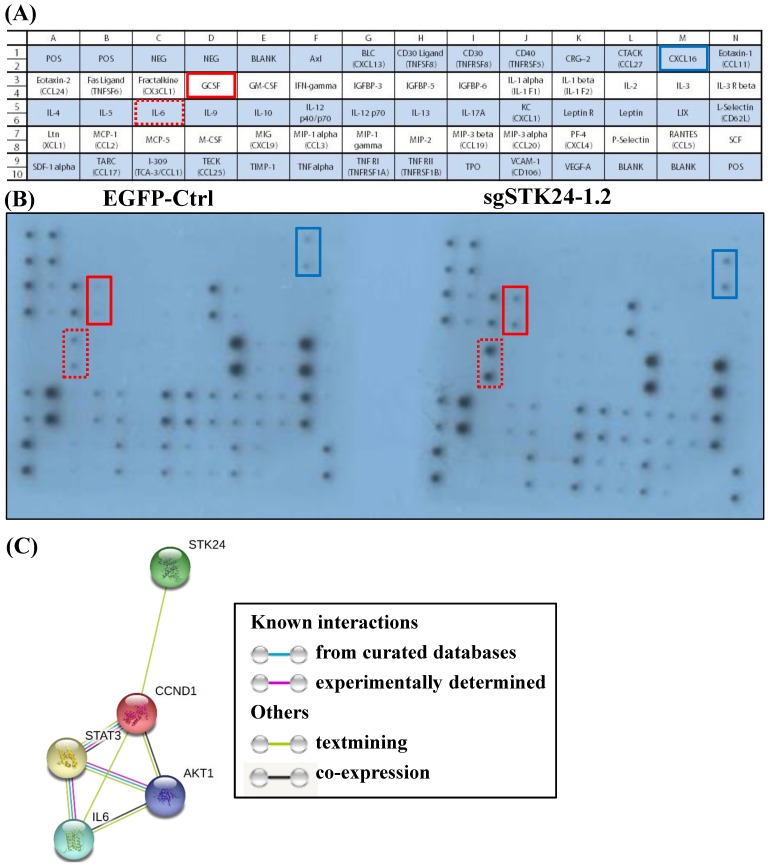
** Cytokine secretion into conditioned medium after STK24 silencing in the M12 cell clones.** (A) Mouse cytokine array maps were used to detect 62 mouse proteins in the cell culture media. (B) The results of a cytokine array of conditioned media from control-pEGFP (left panel) and sgSTK24-RNA1.2 cells (right panel) after 48 h in culture are shown. (C) The protein interaction network of STK24, IL-6, STAT3, CCND1 and AKT1 is shown. The colored lines between the proteins indicate the various lines of evidence that demonstrate the interaction. The evidence for these interactions is derived from both experimental evidence (purple lines) and text-mining evidence (green lines). STK24: serine/threonine-protein kinase 24; STAT3: signal transducer and activator of transcription 3; IL-6: interleukin 6; CCND1: cyclin D1; AKT1: RAC-alpha serine/threonine-protein kinase.

We hypothesized that STK24 inhibits the secretion of pro-inflammatory cytokines by cancer cells. Given the importance of a functional immune system for the effects of STK24 on tumor formation, we further investigated whether cancer cell-secreted cytokines were involved in the tumor microenvironment. Conditioned media from control-pEGFP and sgMST3-RNA1.2 cell cultures were collected after serum-deprivation for 48 h, and the cytokine profiles, which consisted of 62 different factors, were studied (Fig. 8A). Specifically, the cytokine assay of the conditioned media shows that the secretion of interleukin-6 (IL-6), granulocyte colony- stimulating factor (GCSF) and chemokine (C-X-C motif) ligand 16 (CXCL16) were increased by the sgSTK24-1.2 cells compared with the cytokine secretion by the control-pEGFP cells (Fig. 8B). The expression of STK24 alters cytokine/ chemokine secretion in gastric cancer cells. To utilize the protein interaction data that may indicate significant protein interactions in gastric cancer, relevant protein-protein interaction information was retrieved from the Search Tool for the Retrieval of Interacting Genes (STRING) database website (Fig. 8C). The STRING database website reveals a highly-interconnected network and predicts the protein-protein interactions derived from many sources, such as high-throughput experiments, coexpression data, and literature reports. Evidence for the physical interactions between STK24, AKT1, IL-6, CCND1 and STAT3 was found in the STRING database when querying STK24 and IL-6; this evidence was from text mining.

## Discussion

We have successfully established an orthotopic gastric cancer model in C57BL/6 mice. The cell line is named M12 and was deposited at Bioresource Collection and Research Center of Taiwan on Sep. 13, 2016 (Patent No. I604054). This model can be applied to immunotherapy, gene therapy, determining the mechanism of tumor metastasis and drug screening. C57BL/6 mice are an inbred mouse strain and have been favored by researchers for improving the reliability and reproducibility of animal-based experiments 23. Specifically, C57BL/6 mice have been used in antitumor activity and immunological studies 23. Mouse cancer cell lines have been established, and the immunogenicity of these cell lines was evaluated for immune responses to therapies. The most common cell lines, such as MC38 for colon cancer, B16 for melanoma and EL4 for lymphoma, were all generated in mice with a C57BL/6 background. C57BL/6 inbred mice were developed for tumor and immunological studies. We developed this orthotopic mouse model to be a useful tool for the *in vivo* studies of tumorigenesis and the metastasis of gastric cancer, especially for those using genetically engineered mice with a C57BL/6 background.

Germinal center kinases (GCKs) belong to the sterile 20 (STE20)-like kinase family. The GCK family includes the GCK-I, GCK-II, GCK-III, GCK-VI and GCK-VIII subfamilies. STK24 belongs to the GCKIII subfamily. STK24 is involved in apoptosis, cell polarity and migration 24,25. STK24 is expressed in normal and gastric cancer tissues. In a previous study, the location of STK24 expression was examined in the normal, intestinal metaplasia, and tumor tissues of the stomach by immunohistochemistry 12. Advanced gastric cancer cases, such as those with a greater number of tumors and a higher occurrence of STK24 expression, had higher MST3 expression levels 12. In the current study, analyses with the Kaplan-Meier Plotter and Oncomine databases revealed that the downregulation of STK24 is a predictor for a poor prognosis for gastric cancer patients (Fig. 3). Western blot analysis showed the band and the molecular size of the STK24 protein. The quantitative evaluation of the STK24 protein was performed in whole tissues, including cancer cells, stromal tumor-infiltrating cells, and blood cells (Fig. 4). We are aware of the possibility that some STK24 protein may exist in the stromal tumor-infiltrating cells of tumor biopsies and surgical specimens and that this protein may be detected by western blot analyses. The abundance and character of MDSCs and the level of STK24 protein expression may cause clinicopathological features, especially in more aggressive gastric cancer cases. In the current study, it is not clear whether the expression levels of the STK24 protein are associated with the MDSCs of tumor biopsies and surgical specimens in patients with gastric cancer.

In present study, the suppression of STK24 did not affect the cell proliferation of M12 cancer cell *in vitro*. However, the knockdown of STK24 induced the tumor growth of gastric cancer *in vivo.* Those results that implications for several possibilities (1) the accumulation of Ly6C^+^ cells of spleen were associated with tumor progression 26,27, (2) IL-6 expression of knockdown of STK24 cancer cells may be associated with macrophage polarization within the gut microenvironment 28, (3) G-CSF is produced by knockdown of STK24 cancer cells in the tumour microenvironment leading to tumour growth and progression 29.

In the current study, we suggest that STK24 plays an immunoregulatory role in gastric cancer. The complex of STK24 and CCM3 in the regulation of ligand-stimulated exocytosis plays an important regulatory role in neutrophil degranulation 10. Therefore, previous studies have implied that STK24 plays an important role in immune regulation, but the regulatory mechanisms of gastric cancer are incompletely understood. To the best of our knowledge, this is the first study to demonstrate that the downregulation of STK24 expression enhances tumor growth in gastric cancer and that STK24 has a protective effect during tumorigenesis. We used an orthotopic model of gastric cancer in immunocompetent C57BL/6 mice and in immunocompromised NOD/SCID mice (Fig. 6). The NOD/SCID mice are deficient in adaptive immunity, but these mice have a preserved innate immune system. We compared the tumor growth of the tumors from the pEGFP control (EGFP-Ctrl) cells and those from the sgSTK24 cells. The tumor growth results in the NOD/SCID mice (Fig. 6F) were similar to the tumor growth results in the C57BL/6 mice (Fig. 6C). The STK24 knockdown cells were implanted into NOD/SCID mice separately, and the results showed that STK24 knockdown significantly promoted tumor growth to some extent in T- and B-cell immunity-deficient mice. But less tumor suppression in NOD/SCID mice was observed compared with that in immune-compromised mice which implied that except T and B cells, other immune cells, such as neutrophils, macrophages and NK cells, may participate in immune evasion. Therefore, these results also suggested that STK24 might play a role in the development and accumulation of MDSCs *in vivo*. The cellular mechanism of STK24 of tumor cells remain largely unknown as well as MDSC and their secreted soluble factors and cytokines may be key in promoting the gastric cancer progression.

The immune cells in innate immunity include MDSCs, macrophages and neutrophils. High numbers of MDSCs in patients with gastric cancer, pancreatic cancer, esophageal cancer, lung cancer and other tumors play important roles in immunosuppression 30-32**.** MDSCs express high levels of CD11b (a myeloid lineage marker) and GR1 (a granulocytic marker) in a mouse model 33,34. GR1 is composed of Ly6C (monocytic MDSCs) and Ly6G (granulocytic MDSCs). MDSCs express high levels of both arginase (ARG1) and iNOS and have an increased production of reactive oxygen species (ROS) to mediate T-cell suppression 35,36. The inflammatory monocytes (CD11b^+^Ly6C^+^ cells) were recruited to the sites with chronic inflammation and tumors. Different tumor microenvironments contain a functionally distinct subsets of macrophages that are derived from Ly6C^high^ monocytes. Bone marrow-derived Ly6C^high^ monocytes are selectively recruited to injured kidneys and differentiate into functionally distinct populations. In the current study, the silencing of STK24 expression in gastric cancer cells increased the infiltration of CD11b^+^Ly6C^+^ MDSCs and promoted tumorigenesis in orthotopic gastric cancer (Fig. 7). The CD11b^+^Ly6C^+^ cells from the spleens of tumor-bearing animals were investigated, and the results indicate that the CD11b^+^Ly6C^+^ cells were immunosuppressive cells.

Tumor-derived factors activate MDSCs and increase the recruitment of MDSCs in a premetastatic niche 37. The mechanisms of MDSC-facilitated metastasis can be classified into immunosuppressive and immune-independent mechanisms. The immune-independent mechanisms of MDSCs include the enhancement of angiogenesis, extracellular matrix degradation, tumor cell invasion, and the formation of the premetastatic niche 38-40. The immunosuppressive effects of MDSCs include the inhibition of CD8^+^ T cells, NK cells, dendritic cells and macrophages in gastric metastasis. Therefore, MDSCs are key therapeutic targets. The induction and infiltration of MDSCs in the tumor microenvironment are influenced by cytokines/chemokines.

Macrophages are divided into two phenotypes: M1 and M2. M1 macrophages have immune stimulation properties and cytotoxic functions against tumor cells 41. The CD163^+^ and CD11b^+^ tumor-associated macrophages (TAMs) have been associated with a poor prognosis for gastric cancer patients, and the increased number of M1 macrophages may be associated with a better OS for gastric cancer patients 42,43. TAMs play a critical role in promoting tumor progression and metastasis 44,45. A previous study has demonstrated that TAM accumulation in cancer is associated with a poor clinical prognosis and cancer cell drug resistance 46. TAMs also promote tumor cell motility 47. Strategies to enhance cancer therapy will need to convert the pro-tumorigenic M2 phenotype into the anti-tumorigenic M1 TAM phenotype. In addition, STK24 is a potential regulator that may function in the differentiation of monocytes into M1 and M2 macrophages.

In the current study, the secretion of interleukin-6 (IL-6), granulocyte colony-stimulating factor (GCSF) and chemokine (C-X-C motif) ligand 16 (CXCL16) was increased by the STK24-knockdown tumor cells in comparison to the secretion by the control-pEGFP tumor cells (Fig. 8). IL-6 expression is markedly associated with STAT3 and enhances the invasion of gastric cancer cells 48,49. G-CSF-producing gastric tumors are detected at an advanced stage and are associated with a poor prognosis by stimulating tumor proliferation, migration, and angiogenesis 50,51. CXCL16 is highly expressed in many cancers, including ovarian, breast, prostate, colon, and liver cancers, and the expression of CXCL16/CXCR6 correlates with lymph node metastasis in epithelial ovarian carcinoma 52,53. The number of MDSCs rapidly increases in B16F10-bearing mice with tumor recurrence 54. MDSCs are also involved in the promotion of angiogenesis, tumor invasion, and metastases 55,56. MDSC expansion is induced by stem cell factor (SCF), granulocyte colony stimulating factor (GCSF), macrophage colony stimulating factor (MCSF), granulocyte macrophage colony stimulating factor (GMCSF), vascular endothelial growth factor (VEGF), IL-1, IL-6, TNF-α, IL-13, and IL-10 57-59. These cytokines are mainly produced by tumor cells 60. In the current study, we detected that STK24 inhibits the expansion of CD11b^+^Ly6C^+^ cells and F4/80^+^ macrophages and inhibits the tumorigenicity of gastric cancer. The increased secretion of IL-6 and GCSF by STK24-knockdown tumor cells and the enhanced tumor growth were observed in the current study. These findings suggest that the protective mechanisms of STK24 in gastric cancer may be associated with its effects on suppressing MDSC expansion.

The list of relevant interacting proteins retrieved from the STRING database includes STK24, AKT1, IL-6, CCND1 and STAT3 and is shown in Fig. 8C. To our knowledge, this is the first study to report that STK24 directly mediates IL-6/STAT3/CCND1/AKT1 signaling expression in gastric cancer. These results suggest potential functional components underlying the molecular mechanisms of STK24 and may facilitate a better understanding of the signaling networks involved in gastric tumorigenesis. We used an orthotopic gastric cancer C57BL/6 mouse model to evaluate M12 tumor cells for tumor formation after tumor cell injection. We investigated the levels of MDSCs and other immune cells in this orthotopic gastric cancer model. We propose a novel strategy for the treatment of gastric cancer by enhancing the levels of the STK24 protein; the increased levels of STK24 will reduce the number of MDSCs and will therefore reduce their activity.

## Abbreviations

CCND1: Cyclin D1
CRISPR: Clustered regularly interspaced short palindromic repeats
CXCL16: Chemokine (C-X-C motif) ligand 16
GCK: Germinal center kinase
GCSF: Granulocyte colony stimulating factor
GM-CSF: Granulocyte macrophage colony stimulating factor
IL-6: Interleukin-6
MCSF: Macrophage colony stimulating factor
MDSC: Myeloid-derived suppressor cell
MNU: N-nitro-N- methylurea
MST-3: Mammalian STE20-like protein kinase 3
NOD/SCID: Nonobese diabetic/severe combined immunodeficient
PAM: Protospacer adjacent motif
SCF: Stem cell factor
STK24: Serine/threonine-protein kinase 24
STRING: Search tool for the retrieval of interacting genes
TAM: Tumor-associated macrophage
VEGF: Vascular endothelial growth factor
ZD: Zinc-deficient
